# Supplementary material for: Integrated QTL Mapping, Meta-Analysis, and RNA-Sequencing Reveal Candidate Genes for Maize Deep-Sowing Tolerance
Source: Int J Mol Sci. 2023 Apr 5;24(7):6770. doi: 10.3390/ijms24076770 (PMC10094843; doi:10.3390/ijms24076770)
Supplement: Supplementary file 1 [file ijms-24-06770-s001.zip › ijms-2300221-supplementary.pdf]

Table S1. QTLs for six deep-sowing tolerant traits in the $F_{2:3}$ population by single-environment mapping with composite interval mapping (CIM).

Trait	QTL	Bin	Sowing depth	QTL position		LOD	Additive (a)	Dominance (d)	Gene action		PVE (%)
				cM	Marker interval				∣d/a∣	Type	
RAT	qRAT–Ch.1–1	1.08	SW3	93.7	umc1085–umc2181	4.98	–0.16	0.01	0.06	A	4.35
			SW15	93.2	umc1085–umc2181	6.22	–0.21	0.03	0.14	A	5.37
			SW20	93.7	umc1085–umc2181	5.91	–0.18	–0.03	0.17	A	4.98
	qRAT–Ch.3–1	3.00–3.01	SW15	2.1	umc2108–umc2376	5.21	0.24	–0.27	1.13	D	4.74
	qRAT–Ch.5–1	5.04–5.05	SW3	101.9	umc2298–umc1482	6.97	0.11	0.02	0.18	A	6.50
			SW15	101.9	umc2298–umc1482	8.03	–0.28	–0.04	0.14	A	7.31
			SW20	101.9	umc2298–umc1482	7.45	0.25	0.03	0.12	A	6.83
	qRAT–Ch.7–1	7.06	SW3	3.4	umc1760–phi116	11.03	–0.29	0.05	0.17	A	10.12
			SW15	3.1	umc1760–phi116	8.84	–0.13	–0.01	0.08	A	8.18
			SW120	3.6	umc1760–phi116	12.81	–0.17	–0.02	0.12	A	11.96
MESL	qMESL–Ch.1–1	1.09	SW3	110.9	umc2047–bnlg1597	3.10	0.25	0.03	0.12	A	2.89
			SW15	110.8	umc2047–bnlg1597	8.84	–0.75	–0.11	0.10	A	8.34
			SW20	110.8	umc2047–bnlg1597	10.43	–0.91	–0.15	0.16	A	10.01
	qMESL–Ch.1–2	1.10	SW15	145.2	umc1534–umc2223	13.96	–1.17	0.10	0.09	A	13.12
	qMESL–Ch.3–1	3.04	SW15	55.3	umc1527–umc2261	7.10	–1.88	–0.21	0.11	A	11.60
			SW20	56.1	umc1527–umc2261	4.77	–1.03	–1.20	1.17	D	4.94
	qMESL–Ch.4–1	4.06–4.07	SW3	88.1	umc1869–umc1775	4.08	–0.34	–0.02	0.06	A	3.81
			SW15	88.0	umc1869–umc1775	10.02	–0.83	–0.09	0.11	A	9.46
			SW20	88.2	umc1869–umc1775	14.84	-1.25	-0.18	0.14	A	13.97
	qMESL–Ch.4–2	4.08	SW15	119.1	umc1612–umc1313	4.44	–1.10	0.08	0.07	A	6.98
			SW20	119.4	umc1612–umc1313	4.61	0.17	0.10	0.59	PD	8.42
	qMESL–Ch.6–1	6.01	SW3	14.3	umc2311–umc2196	9.30	–1.01	–1.51	1.50	OD	8.11
			SW15	14.3	umc2311–umc2196	5.81	–0.61	0.97	1.59	OD	5.06
			SW20	14.3	umc2311–umc2196	11.45	–1.14	–1.43	1.25	OD	10.03
	qMESL–Ch.7–1	7.02	SW3	67.0	umc1585–umc2526	3.08	–0.80	–0.03	0.04	A	4.93
			SW15	66.9	umc1585–umc2526	2.29	–0.61	0.08	0.13	A	3.67
COLL	qCOLL–Ch.1–1	1.09	SW3	111.2	umc2047–bnlg1597	7.43	0.21	0.03	0.14	A	12.15
			SW15	111.0	umc2047–bnlg1597	6.36	0.19	–0.01	0.05	A	10.38
			SW20	111.4	umc2047–bnlg1597	8.61	0.28	–0.04	0.14	A	14.12
	qCOLL–Ch.3–1	3.08	SW3	114.1	phi088–umc2174	3.65	0.79	0.84	1.06	D	3.82
			SW20	114.1	phi088–umc2174	11.24	1.24	–1.58	1.27	OD	9.89
	qCOLL–Ch.4–1	4.08–4.09	SW20	130.5	umc1313–umc2287	5.51	–0.14	0.01	0.07	A	9.07
	qCOLL–Ch.9–1	9.05	SW15	102.3	umc2343–umc2341	3.23	0.11	–0.06	0.55	PD	5.83

			SW20	102.3	umc2343–umc2341	3.09	–0.09	0.03	0.33	PD	5.50
	qCOLL–Ch.10–1	10.04–10.07	SW15	126.4	umc1507–bnlg1185	3.00	–0.63	–0.71	1.13	D	3.02
			SW20	126.9	umc1507–bnlg1185	3.03	0.65	0.71	1.09	D	3.08
MESL+COLL	qMESL+COLL–Ch.1–1	1.05–1.06	SW3	80.6	umc1076–umc1748	6.17	–0.18	–0.02	0.11	A	10.06
			SW15	81.0	umc1076–umc1748	7.39	–0.24	0.01	0.04	A	12.15
			SW20	81.0	umc1076–umc1748	4.91	–0.16	–0.12	0.75	PD	8.93
	qMESL+COLL–Ch.1–2	1.09	SW15	110.9	umc2047–bnlg1597	3.50	0.10	0.01	0.10	A	5.80
			SW20	111.0	umc2047–bnlg1597	5.68	0.18	–0.03	0.17	A	9.37
	qMESL+COLL–Ch.2–1	2.00–2.01	SW3	11.3	umc1419–phi96100	4.08	–0.89	1.00	1.12	D	4.13
			SW15	10.9	umc1419–phi96100	8.92	–1.91	–1.98	1.04	D	9.05
			SW20	11.0	umc1419–phi96100	10.75	–2.30	2.21	0.96	D	11.01
	qMESL+COLL–Ch.4–1	4.08	SW3	120.1	umc1612–umc1313	3.03	–0.64	–0.23	0.36	PD	5.54
			SW15	120.3	umc1612–umc1313	9.21	–1.97	2.00	1.02	D	9.40
			SW20	120.3	umc1612–umc1313	3.97	–0.88	–0.79	0.90	D	4.08
	qMESL+COLL–Ch.6–1	6.01	SW15	14.9	umc2311–umc2196	6.54	0.73	1.01	1.38	OD	5.81
			SW20	14.9	umc2311–umc2196	9.06	1.00	–1.34	1.34	OD	8.15
	qMESL+COLL–Ch.9–1	9.00–9.01	SW20	54.7	umc2393–phi028	5.00	0.59	0.88	1.49	OD	4.49
	qMESL+COLL–Ch.10–1	10.02–10.03	SW15	85.9	umc2034–umc2349	3.08	–0.63	0.60	0.95	D	3.16
			SW20	85.9	umc2034–umc2349	6.16	1.27	1.11	0.87	D	6.30
	qMESL+COLL–Ch.10–2	10.04	SW15	122.2	umc1077–umc1678	3.88	0.79	0.82	1.04	D	3.97
			SW20	122.2	umc1077–umc1678	9.89	–1.18	1.60	1.36	OD	8.92
MESL/COLL	qMESL/COLL–Ch.4–1	4.08	SW3	120.0	umc1612–umc1313	8.98	–0.98	1.11	1.13	D	8.43
			SW15	120.0	umc1612–umc1313	10.27	–1.12	–1.09	0.97	D	9.56
			SW20	120.0	umc1612–umc1313	11.27	–1.07	–1.19	1.11	D	10.69
	qMESL/COLL–Ch.8–1	8.03	SW15	95.3	bnlg1460–umc1289	6.24	–0.84	0.55	0.65	PD	5.88
			SW20	95.6	bnlg1460–umc1289	7.93	–0.94	–0.49	0.52	PD	7.35
	qMESL/COLL–Ch.9–1	9.04	SW3	85.2	umc2121–umc1387	5.94	–1.03	–1.16	1.13	D	5.59
			SW20	85.5	umc2121–umc1387	8.99	–1.16	–1.01	0.87	D	8.50
SDL	qSDL–Ch.1–1	1.03–1.04	SW15	44.6	umc1770–bnlg2204	3.87	–1.03	–0.63	0.61	PD	6.58
	qSDL–Ch.1–2	1.06–1.08	SW3	88.0	umc1748–umc1085	7.87	–1.62	–0.79	0.49	PD	12.21
			SW15	88.0	umc1748–umc1085	3.14	–0.66	0.42	0.64	PD	4.87
	qSDL–Ch.1–3	1.08–1.09	SW15	106.4	umc2240–umc2047	6.93	–1.44	–0.5	0.49	PD	10.75
			SW20	106.6	umc2240–umc2047	5.35	1.10	–1.21	1.10	D	5.48
	qSDL–Ch.5–1	5.03–5.04	SW3	77.9	umc1048–umc1860	4.48	–1.24	–0.16	0.13	A	7.33
			SW20	77.7	umc1048–umc1860	3.60	1.89	0.94	0.50	PD	6.04
	qSDL–Ch.7–1	7.02	SW3	70.0	umc1585–umc2526	3.85	1.09	0.10	0.09	A	6.26
			SW15	70.0	umc1585–umc2526	6.71	1.86	0.11	0.06	A	10.91

		SW20	70.0	umc1585–umc2526	8.70	2.45	0.18	0.07	A	14.17
qSDL–Ch.8–1	8.06–8.07	SW3	142.7	umc2711–bnlg1828	3.25	1.69	–0.57	0.34	PD	5.49
		SW15	141.9	umc2711–bnlg1828	4.01	2.06	–1.44	0.70	PD	6.75
		SW20	142.3	umc2711–bnlg1828	4.66	2.39	–1.62	0.68	PD	7.84

RAT, emergence rate; MESL, mesocotyl length; COLL, coleoptile length; MESL+COLL, total length between mesocotyl and coleoptile, MESL/COLL, length ratio of mesocotyl to coleoptile; SDL, seedling length; SW3, SW15, and SW20 indicate the sowing depths of 3cm, 15cm, and 20cm, respectively; LOD, logarithm of odds; additive (a) effect positive values indicate that K12 carries the allele for an increase in corresponding deep-sowing trait, whereas negative values indicate that W64A carries the allele and contributes to an increase in corresponding trait; the QTL action [|dominance (d)/additive (a)|; additive (A), |d/a|=0.00~0.20; partial-dominance (PD), |d/a|=0.21~0.80; dominance (D), |d/a|=0.81~1.20; over–dominance (OD), |d/a|>1.20]; PVE, phenotypic variance explained by the QTL.

Table S2. The functional annotations of corresponding candidate genes in 13 major QTLs and meta-QTLs (MQTLs) regions.

**Candidate genes**	**Encoded protein**	**Gene ontology (GO) annotation**
GRMZM2G065635	Brassinosteroid synthesis 1	response to brassinosteroid (GO:0009741); brassinosteroid biosynthetic process GO:0016132); brassinosteroid homeostasis (GO:0010268); jasmonic acid mediated signaling pathway (GO:0009867); leaf development (GO:0048366)
GRMZM2G124532	PhytochromeB1 (PHYB1)	photoreceptor activity (GO:0009881); detection of visible light (GO:0009584); red, far-red light phototransduction (GO:0009585)
GRMZM2G326335	Phenylalanine ammonia lyase homolog 10 (PAL10)	L-phenylalanine catabolic process (GO:0006559); cinnamic acid biosynthetic process (GO:0009800); phenylalanine ammonia-lyase activity (GO:0045548)
GRMZM2G040638	Peroxidase (POD)	peroxidase activity (GO:0004601); regulation of circadian rhythm (GO:0042752);plant-type cell wall (GO:0009505)
GRMZM2G107499	Protein LNK1	circadian rhythm (GO:0007623); regulation of transcription, DNA-templated (GO:0006355)
GRMZM2G403620	Protein rough sheath 2 (RS2)	response to auxin (GO:0009733); response to gibberellin (GO:0009739); response to salicylic acid (GO:0009751); response to jasmonic acid (GO:0009753); leaf morphogenesis (GO:0009965); leaf formation (GO:0010338)
GRMZM2G422419	Auxin-responsive protein SAUR71	response to auxin (GO:0009733)
GRMZM2G419267	Beta-2-xylosyltransferase (xt1)	UDP-xylosyltransferase activity (GO:0035252); xylosyltransferase activity (GO:0042285); glycoprotein 2-beta-D-xylosyltransferase activity (GO:0050513); shoot system development (GO:0048367)
GRMZM2G404443	Cytokinin dehydrogenase 6 precursor	cytokinin metabolic process (GO:0009690); cytokinin dehydrogenase activity (GO:0019139)
GRMZM2G167986	Cytochrome P450 CYP78A1	monooxygenase activity (GO:0004497); oxidoreductase activity (GO:0016491); regulation of timing of plant organ formation (GO:0090709)
GRMZM2G103773	Brassinosteroid-deficient dwarf 1	microtubule bundle formation (GO:0001578); monooxygenase activity (GO:0004497); brassinosteroid homeostasis (GO:0010268); brassinosteroid biosynthetic process(GO:0016132)
GRMZM2G033871	Auxin responsive protein (SAUR)	response to auxin (GO:0009733)
GRMZM2G146108	Auxin responsive protein (SAUR)	response to auxin (GO:0009733)
GRMZM2G157727	PhytochromeA1 (PHYA1)	photoreceptor activity (GO:0009881); detection of visible light (GO:0009584); red, far-red light phototransduction (GO:0009585)
GRMZM2G057935	PhytochromeC1 (PHYC1)	photoreceptor activity (GO:0009881); detection of visible light (GO:0009584); red, far-red light phototransduction (GO:0009585)

GRMZM2G040736	Cytokinin response regulator 1	phosphorelay signal transduction system (GO:0000160)
GRMZM2G107228	Peroxidase (POD)	peroxidase activity (GO:0004601); response to oxidative stress (GO:0006979); plant-type cell wall (GO:0009505);
GRMZM2G138268	Auxin-responsive protein (IAA10)	auxin-activated signaling pathway (GO:0009734)
GRMZM2G167794	Auxin-responsive protein (IAA11)	auxin-activated signaling pathway (GO:0009734)
GRMZM2G174249	Invertase cell wall 8 (incw8)	hydrolase activity, hydrolyzing O-glycosyl compounds (GO:0004553);
GRMZM2G143328	Transcription factor MYB53	sequence-specific DNA binding (GO:0043565); DNA binding (GO:0003677)
GRMZM2G410499	Auxin responsive protein (SAUR)	response to auxin (GO:0009733)
GRMZM2G130043	Starch synthase V	glycogen (starch) synthase activity (GO:0004373); starch metabolic process (GO:0005982); starch synthase activity (GO:0009011); starch biosynthetic process (GO:0019252)
GRMZM2G003501	3-ketoacyl-CoA synthase	response to cold (GO:0009409); response to light stimulus (GO:0009416)
GRMZM2G015049	Auxin responsive protein SAUR50 (SAUR)	response to auxin (GO:0009733)
GRMZM2G017852	U-box domain-containing protein 44	ubiquitin-protein transferase activity (GO:0004842); abscisic acid biosynthesis (GO:0010115)
GRMZM2G045171	Sucrose synthase 6	sucrose synthase activity (GO:0016157); transferase activity (GO:0016740); sucrose metabolic process (GO:0005985)
GRMZM2G455658	Nana2-like 1	lignin metabolic process (GO:0009808); unidimensional cell growth (GO:0009826); plant-type secondary cell wall biogenesis (GO:0009834); brassinosteroid biosynthetic process (GO:0016132)
GRMZM2G102163	Receptor-like serine/threonine-protein kinase ALE2	protein kinase activity (GO:0004672); cell surface receptor signaling pathways (GO:0007166); protoderm histogenesis (GO:0010068); cell differentiation (GO:0030154); cuticle development (GO:0042335), plant organ formation (GO:1905393),
GRMZM2G102216	Glutathione S-transferase family protein	glutathione transferase activity (GO:0004364); auxin-mediated signaling pathways (GO:0010930)
GRMZM2G475683	SAUR12-auxin-responsive SAUR family member (SAUR)	response to auxin (GO:0009733)
GRMZM2G174834	AP2-EREBP transcription factor	DNA-binding transcription factor activity (GO:0003700)
GRMZM2G460406	L-ascorbate peroxidase 3 peroxisomal	response to reactive oxygen species (GO:0000302); cytochrome-c peroxidase activity (GO:0004130); L-ascorbate peroxidase activity (GO:0016688); hydrogen peroxide catabolic process (GO:0042744)
GRMZM2G361993	Auxin-responsive protein SAUR32 (SAUR)	response to auxin(GO:0009733)
GRMZM2G088212	Catalase 1 (CAT1)	catalase activity (GO:0004096); peroxidase activity (GO:0004601); circadian rhythm (GO:0007623); response to abscisic acid (GO:0009737); response to salicylic acid (GO:0009751)
GRMZM2G059212	Callose synthase 3	1,3-beta-D-glucan synthase activity (GO:0003843); (1->3)-beta-D-glucan biosynthetic process (GO:0006075); regulation of cell shape (GO:0008360); cell wall organization (GO:0071555)
GRMZM2G050705	Glycosyltransferase family protein 28	dolichol-linked oligosaccharide biosynthetic process (GO:0006488); transferase activity (GO:0016740)
GRMZM2G305856	Transcription factor MYB59-like	DNA binding (GO:0003677)

GRMZM2G450233	Peroxidase 5 (POD5)	peroxidase activity (GO:0004601); response to oxidative stress (GO:0006979)
GRMZM2G044358	GA 3-oxidase 2 isoform X1	gibberellin 3-beta-dioxygenase activity (GO:0016707)
GRMZM2G027723	Cellulose synthase 2	plant-type primary cell wall biogenesis (GO:0009833); cellulose synthase activity (GO:0016759); cellulose synthase (UDP-forming) activity (GO:0016760); cellulose biosynthetic process (GO:0030244); cell wall organization (GO:0071555)
GRMZM2G022904	Cytokinin hydroxylase	monooxygenase activity (GO:0004497); oxidoreductase activity, acting on paired donors, with incorporation or reduction of molecular oxygen (GO:0016705); trans-zeatin biosynthetic process (GO:0033466)
GRMZM2G011463	Auxin-responsive SAUR family member (SAUR37)	response to auxin (GO:0009733)
GRMZM2G065928	Abscisic acid 8-hydroxylase 4	multicellular organism development (GO:0007275); brassinosteroid homeostasis (GO:0010268); (+)-abscisic acid 8'-hydroxylase activity (GO:0010295); brassinosteroid biosynthetic process (GO:0016132); abscisic acid catabolic process (GO:0046345)
GRMZM2G112984	Pectinesterase	pectinesterase activity (GO:0030599); cell wall modification (GO:0042545); aspartyl esterase activity (GO:0045330); pectin catabolic process (GO:0045490); pectinesterase inhibitor activity (GO:0046910)
GRMZM2G447271	Laccase 21 (LAC21)	oxidoreductase activity(GO:0016491); lignin catabolic process(GO:0046274)
GRMZM2G336337	Laccase 22 (LAC22)	oxidoreductase activity (GO:0016491); lignin catabolic process (GO:0046274)
GRMZM2G092174	PhytochromeB2 (PHYB2)	detection of visible light (GO:0009584); red, far-red light phototransduction (GO:0009585); photoreceptor activity (GO:0009881)
GRMZM2G126834	ARR-B-transcription factor 1	phosphorelay signal transduction system (GO:0000160); cytokinin-activated signaling pathway (GO:0009736)
GRMZM2G122340	Cytokinin oxidase 11	cytokinin metabolic process (GO:0009690); cytokinin dehydrogenase activity (GO:0019139)

Table S3. Summary of quality preprocessing of RNA-Sequencing (RNA-Seq) data in both parents (W64A and K12).

Sample code	Raw Date Size (bp)	Clean Date Size (bp)	Clean Date Rate (%)	Total Reads	Q20 percentage (%)	Q30 percentage (%)	GC (%)	Mapped Reads	Mapping Rate (%)
K12–MES(SW3)	9280368041	7852391550	84.53	55602589	97.7	93.45	53.63	48326857	86.81
K12–MES(SW20)	9114478837	6954957300	76.32	52798507	98.14	94.52	53.29	47129827	89.27
W64A–MES(SW3)	7010755947	6941989700	100.00	47020862	98.23	94.76	53.91	42455639	90.43
W64A–MES(SW20)	8190515323	8192650400	99.56	47635087	98.23	94.77	54.72	42527764	89.33
K12–COL(SW3)	7962591997	6660647300	84.73	46565669	98.1	94.46	54.61	42068030	90.28
K12-COL(SW20)	8351880567	8315574300	99.25	53620385	98.17	94.63	54.9	47996023	89.58
W64A–COL(SW3)	7735085100	7252831295	89.37	51232339	98.09	94.4	53.6	46106931	90.24
W64A–COL(SW20)	7222181400	6834207251	90.46	45931682	98.16	94.58	54.67	41423237	90.24
Average	8108482152	7375656137	90.58	50050890	98.1	94.45	54.17	44754288	89.52

MES, mesocotyl; COL, coleoptile; SW3 and SW20 indicate the sowing depth environment of 3cm and 20cm, respectively.

Table S4. Sequences of primers used in quantitative real-time PCR (qRT-PCR) analysis.

Gene ID	Forward primer (5' to 3')	Reverse primer (5' to 3')
GRMZM2G126010 (Actin–1)	CGATTGAGCATGGCATTGTCA	CCCACTAGCGTACAACGAA
GRMZM2G157727	ATGAACTCGATCGCATCCCT	CACATATCTGGGGCTCTCGT
GRMZM2G045171	ATGGACCACAGGTATCATTTCTCA	TGGCATTGTGAACGCATAGTG
GRMZM2G017852	TCCTCAAGTGGTTCAGGGAGT	CAGGTCAGCTATGGTGGGTATG
GRMZM2G450233	TGACCAACGCAACCATGAAG	TCCTGATGAACCTGGCTGAC
GRMZM2G088212	CCGAATCCAAAGACCAAT	ATGCCAACATCGTCAAAGAG

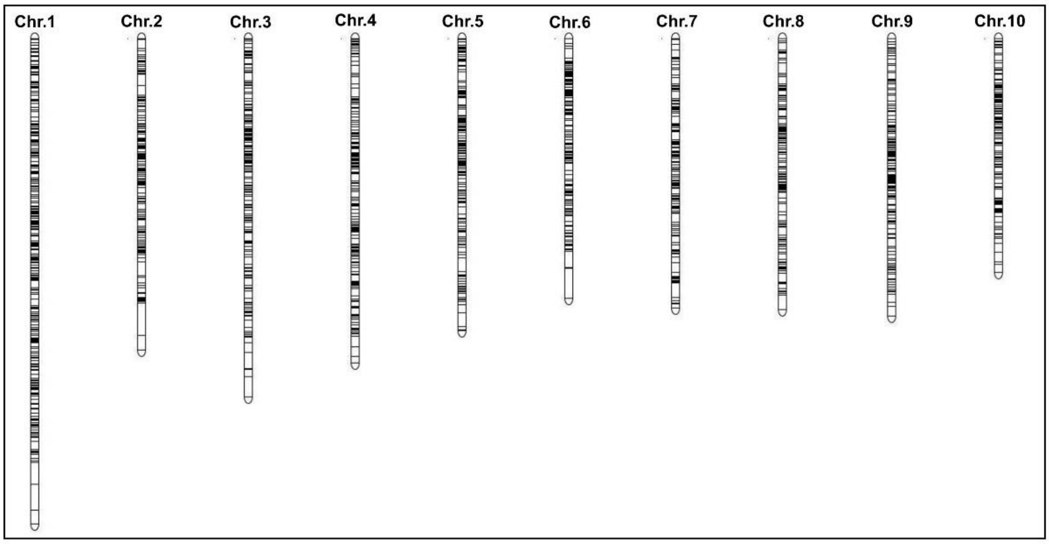

Figure S1. Consensus map constructed across three original mapping datasets.
